# Dabigatran for the Treatment and Secondary Prevention of Venous Thromboembolism; A Cost-Effectiveness Analysis for the Netherlands

**DOI:** 10.1371/journal.pone.0163550

**Published:** 2016-10-24

**Authors:** J. Stevanović, L. A. de Jong, B. S. Kappelhoff, E. P. Dvortsin, M. Voorhaar, M. J. Postma

**Affiliations:** 1 Unit of PharmacoEpidemiology & PharmacoEconomics (PE2), University of Groningen, Groningen, the Netherlands; 2 Boehringer Ingelheim, Alkmaar, the Netherlands; 3 Institute for Science in Healthy Aging & healthcaRE (SHARE), University Medical Center Groningen (UMCG), University of Groningen, Groningen, the Netherlands; 4 Department of Epidemiology, UMCG, University of Groningen, Groningen, the Netherlands; Macau University of Science and Technology, MACAO

## Abstract

**Background:**

Dabigatran was proven to have similar effect on the prevention of recurrence of venous thromboembolism (VTE) and a lower risk of bleeding compared to vitamin K antagonists (VKA). The aim of this study is to assess the cost-effectiveness (CE) of dabigatran for the treatment and secondary prevention in patients with VTE compared to VKAs in the Dutch setting.

**Methods:**

Previously published Markov model was modified and updated to assess the CE of dabigatran and VKAs for the treatment and secondary prevention in patients with VTE from a societal perspective in the base-case analysis. The model was populated with efficacy and safety data from major dabigatran trials (i.e. RE-COVER, RECOVER II, RE-MEDY and RE-SONATE), Dutch specific costs, and utilities derived from dabigatran trials or other published literature. Univariate, probabilistic sensitivity and a number of scenario analyses evaluating various decision-analytic settings (e.g. the perspective of analysis, use of anticoagulants only for treatment or only for secondary prevention, or comparison to no treatment) were tested on the incremental cost-effectiveness ratio (ICER).

**Results:**

In the base-case scenario, patients on dabigatran gained an additional 0.034 quality adjusted life year (QALY) while saving €1,598. Results of univariate sensitivity analysis were quite robust. The probability that dabigatran is cost-effective at a willingness-to-pay threshold of €20,000/QALY was 98.1%. From the perspective of healthcare provider, extended anticoagulation with dabigatran compared to VKAs was estimated at €2,158 per QALY gained. The ICER for anticoagulation versus no treatment in patients with equipoise risk of recurrent VTE was estimated at €33,379 per QALY gained. Other scenarios showed dabigatran was cost-saving.

**Conclusion:**

From a societal perspective, dabigatran is likely to be a cost-effective or even cost-saving strategy for treatment and secondary prevention of VTE compared to VKAs in the Netherlands.

## Introduction

Venous thromboembolism (VTE) can manifest as deep vein thrombosis (DVT) and/or pulmonary embolism (PE) [1]. The health burden associated with VTE is mostly determined with the risk of a fatal PE and risk of considerable long-term morbidity associated with the development of post thrombotic syndrome (PTS) or chronic thromboembolic pulmonary hypertension (CTEPH). Moreover, recurrent DVT (rDVT) occurs in approximately 7% of patients per year and reaches to about one quarter to one third of patients within 8 years [2,3]. The health related quality of life (HRQoL) in VTE patients is also affected. For example, a Dutch study found distinctly lower HRQoL scores measured with SF-36 questionnaires in patients with PE compared to the general population on the subscales: social functioning, emotional, general health, physical and vitality [4].

In the Netherlands, the DVT incidence was estimated at approximately 16,000 to 20,000 cases per year [5]. Though, the overall incidence of PE in the Netherlands is unknown, a survey among Dutch pulmonologists/internists indicated an incidence of suspected PE at 2.6 per 1,000 patients per year [2], while in general practice, 0.2 PEs per 1,000 patients were reported [6].

Both national and international guidelines recommend anticoagulation therapy as an effective measure to prevent thrombus propagation and recurrence in VTE patients [1,2]. For the initial treatment phase of VTE, low-molecular-weight heparins (LMWHs) for at least 5 days combined with subsequent administration of vitamin K antagonists (VKAs; e.g. warfarin, acenocoumarol or phenprocoumon), or rivaroxaban are recommended. For the maintenance phase, the use of VKAs or rivaroxaban is recommended for at least three months [1,7]. The need to continue anticoagulation should be re-assessed in patients based on individual patients’ risk-benefit balance every three months as there is no strong nor clear differentiation between treatment and prevention phases [1,7].

VKAs present a highly effective anticoagulation treatment with low acquisition costs and conventional bleeding management. However, their use is limited by a narrow therapeutic range as defined by the international normalised ratio (INR) between 2.0 and 3.0 and several interactions with other drugs and food. To achieve the anticoagulant effect inside the required INR range, regular monitoring and dose-adjustment is required for treatment with VKAs. In the Dutch healthcare system, INR-monitoring is handled by thrombotic services or patient self-management. Though it is considered highly effective in the Netherlands, INR-monitoring of course directly affects expenditures from both healthcare provider and societal perspectives. In particular, next to the costs of material, labour, nurse visits, training and material for self-management, there are various out-of-the pocket expenses (for example travel costs of patients) and productivity loss costs associated with monitoring visits impacting the broader societal economic burden.

Recently, in the RE-COVER and RE-COVER II trials, dabigatran, a novel oral anticoagulant (NOAC) was shown to have similar effect on VTE recurrence and a lower risk of for clinically relevant non-major bleeding (CRNMB) and for any bleeding compared to VKA [8,9]. When administered for the extended treatment in patients with VTE who had completed at least three months of initial therapy, dabigatran was non-inferior in preventing rVTE events and showed a better safety profile than VKA (the RE-MEDY trial) in high risk patients (increased risk for rVTE), but a significantly better efficacy in preventing rVTE and higher risk of bleedings than placebo (the RE-SONATE trial) [10].

Importantly, both health and economic consequences associated with the use of dabigatran compared to VKAs need to be considered when choosing the optimal treatment strategy. A formal pharmacoeconomic comparison of the two anticoagulant treatments should be conducted to account for all the relevant health consequences such as likelihood of rVTE, bleedings, PTS, CTEPH, death and other adverse events, as well as all relevant cost parameters including the costs of drugs, administration, INR-monitoring, event-related costs and various indirect costs.

The aim of this study is to assess the cost-effectiveness (CE) of dabigatran for the treatment and secondary prevention in high risk patients of DVT and PE compared to VKAs for the Dutch situation.

## Methods

### Decision model

**Fig 1:**
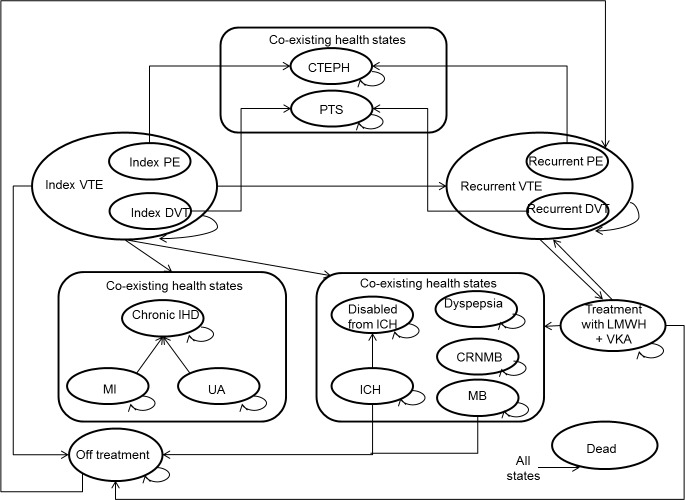
Markov model. VTE, venous thromboembolism; DVT, deep vein thrombosis; PE, pulmonary embolism; r, recurrent; LMWH, low molecular weight heparin; CRNMB = clinically relevant non-major bleed event; ICH = intracranial haemorrhage; MB = major bleed; MI = myocardial infarction; CTEPH = chronic thromboembolic pulmonary hypertension; PTS = post thrombotic syndrome; UA, unstable angina; IHD, ischemic heart disease.

A previously published Markov model was modified and updated to assess the CE of dabigatran and VKAs for the treatment and secondary prevention of DVT and PE in the Dutch setting [11]. The health states included in the model were: index VTE, rVTE, major or clinically relevant bleeding (MCRB), CTEPH, PTS, other adverse events (i.e. myocardial infarction (MI), unstable angina (UA) and dyspepsia), off-treatment and death from other causes (Fig 1).

In the *base-case*, the use of dabigatran was compared to VKAs for up to 6 months of treatment followed by up to 18 months of secondary prevention in high risk patients. The flow of patients with an index VTE event through the Markov model is detailed elsewhere [11]. The proportion of high risk in patients with VKA or dabigatran were balanced via randomization in the study and kept the same in the model. Shortly, at the start of the simulation, a hypothetical cohort of 10,000 adult patients (mean age 54.7 years [8,9]) for whom at least 6 months of anticoagulant therapy was considered appropriate entered the model following an index VTE (i.e. index DVT or index PE) event and received initial treatment with LMWHs followed by either dabigatran or VKAs. The duration of treatment with LMWHs was assumed to be 5 days in the dabigatran treatment arm following the summaries of product characteristics (SCPs) for dabigatran, and 9 days in the VKAs arm in line with the RE-COVER trials. Patients in the index VTE state were exposed to the risk of rVTE, MCRB, CTEPH, PTS, other adverse events, treatment discontinuation and death from other causes. After the initial 6 months of treatment, patients who remained in the index health state were simulated to receive up to 18 months of anticoagulants for secondary prevention, reflecting patient profiles from the RE-MEDY trial [10].

*rVTE* could occur in any model cycle, however, the model was restricted to a maximum of two rVTEs [12]. Furthermore, a distinction was made between different forms of rVTE: fatal VTE, non-fatal PE, proximal DVT and distal DVT. After a first rVTE, patients from both treatment arms were assumed to stop the initial treatment and initiate or reinitiate a 6 months standard treatment course of LMWHs, followed by VKAs.

For patients experiencing a *MCRB*, a distinction was made between an intracranial haemorrhage (ICH), other major bleed (MB), and a CRNMB. We did not differentiate between high and low risk MCRB, as the clinical trial populations were balanced via randomization in terms of baseline bleeding risks. Regarding the VTE risk, we refer to the inclusion criteria for RECOVER, where planned treatment duration should be at least 6 months; that allows to assume that the same patient population can continue to be treated for a longer period [8,9]. Subsequently, the calibration of incidences was not applied, as low risk groups were tested in a separate scenario. If ICH occurred, in the model it could lead to permanent disability, death, or recovery. MBs were modelled to lead to death or recovery. Furthermore, it was assumed that patients can experience up to two major bleeds (ICH or MB) during the entire time they may spend on anticoagulation; one event could be experienced during treatment phase with study medication and one event during LMWHs/VKAs re-treatment [12]. CRNMB could occur at every model cycle while on anticoagulation [12]. After a MB or ICH, all patients were assumed to discontinue treatment altogether having no further risk of bleeding, but continuing to be exposed to a risk of rVTE. *Other adverse events* of anticoagulant therapy captured by the model are UA, MI and dyspepsia. Patients with a non-fatal MI or UA could suffer from chronic ischemic heart disease (IHD), or recover. Mortality after IHD was assumed to be part of the population mortality in the model. In the model, all patients who experienced a first or recurrent PE (rPE) were at risk to develop *CTEPH*, while those with an index or rDVT were at risk of *PTS*.

During treatment or secondary prevention phases, all patients could discontinue treatment prior to reaching the maximum planned duration of treatment due to reasons other than rVTE or ICH/MB. If discontinuation occurs, patients move to the *off-treatment* state where they continue to experience a risk of rVTE, but no further risk of bleeds. Finally, patients in any of the health states were at risk to *die from other causes*. Patient movement between health states was modeled using 1-month cycles until death.

The final outcome of the decision model is the incremental cost-effectiveness ratio (ICER) of dabigatran compared to VKAs. Quality-adjusted life-years (QALYs) and life-years (LYs) gained were estimated as a measure of effectiveness. All relevant costs reflect a societal perspective in the base-case analysis and are inflated, if necessary, to price year 2013 using the Dutch consumer price index [13]. Future costs and health effects were discounted by 4% and 1.5% annually after the first year, according to the Dutch guidelines for pharmacoeconomic research [14].

### Transition probabilities

In the base-case, to estimate the transition probabilities between the health states in the model during the treatment and prevention phases, data were used from a published meta-analysis of the RE-COVER and RE-COVER II trials [8,9] and the RE-MEDY trial [10], respectively. This was in line with the previously published Markov model [11].

In particular, the baseline probabilities of rVTE and MCRB were calculated from the observed incidence in the VKA arm of the aforementioned trials. For the treatment phase, the incidences of rVTE and MCRB were log transformed with respect to time, to better reflect the occurrence pattern of these events in the trials. For the secondary prevention phase, the incidences were not varied with time. To calculate the probabilities of events while on dabigatran, the estimated treatment effect (hazard ratio (HR)) for each trial endpoint was applied to the risk in the VKA arm (Table 1).

**Table 1:** Distribution and parameter limits for the transition probabilities in the model as used in the probabilistic sensitivity analysis. CI, confidence interval; r VTE, recurrent venous thromboembolism; MCRB, major or clinically relevant bleeding; VKA, vitamin K antagonists; HR, hazard ratio; D, Dirichlet distribution applying to 2 or 3 linked probabilities with the parameter corresponding to the specific marginal Beta distribution in italics; DVT, deep vein thrombosis; PE, pulmonary embolism; CRNMB = clinically relevant non-major bleed event; ICH = intracranial haemorrhage; MB = major bleed; MI = myocardial infarction; UA, unstable angina; CTEPH = chronic thromboembolic pulmonary hypertension; PTS = post thrombotic syndrome.

Clinical variable	Value	CI (95%)	Distribution	Reference
Incidence of rVTE (baseline risk), treatment	2.43%	-	Beta(α = 62,β = 2492)	[8,9]
Incidence of MCRB (baseline risk), treatment	7.68%	-	Beta(α = 189,β = 2273)	[8,9]
*Treatment effects*				
*Treatment phase*				
rVTE, dabigatran vs VKA (HR)	1.09	0.77–1.54	Normal (log scale)	[8,9]
MCRB, dabigatran vs VKA (HR)	0.56	0.45–0.71	Normal (log scale)	[8,9]
*Secondary prevention*				
rVTE, dabigatran vs VKA (HR)	1.44	0.78–2.64	Normal (log scale)	[10]
rVTE, dabigatran vs placebo (HR)	0.08	0.02–0.25	Normal (log scale)	[10]
MCRB, dabigatran vs VKA (HR)	0.55	0.41–0.72	Normal (log scale)	[10]
MCRB, dabigatran vs placebo (HR)	2.69	1.43–5.07	Normal (log scale)	[10]
*Type of recurrent VTE events*				
*Treatment phase*				
Dabigatran				
Non-fatal PE	33.80%		D(23,43,2)	[8,9]
Proximal DVT	63.20%		D(23,43,2)	[8,9]
VTE-related death	2.90%		D(23,43,2)	[8,9]
VKA				
Non-fatal PE	33.90%		D(21,38,3)	[8,9]
Proximal DVT	61.30%		D(21,38,3)	[8,9]
VTE-related death	4.80%		D(21,38,3)	[8,9]
*Secondary prevention*				
Dabigatran (RE-MEDY trial)				
Non-fatal PE	34.60%		D(9,16,1)	[10]
Proximal DVT	61.50%		D(9,16,1)	[10]
VTE-related death	3.80%		D(9,16,1)	[10]
Dabigatran (RE-SONATE trial)				
Non-fatal PE	33.30%		D(1,2)	[10]
Proximal DVT	66.70%		D(1,2)	[10]
VTE-related death	0.00%		Fixed	[10]
VKA				
Non-fatal PE	22.20%		D(4,13,1)	[10]
Proximal DVT	72.20%		D(4,13,1)	[10]
VTE-related death	5.60%		D(4,13,1)	
After therapy discontinuation				
Non-fatal PE	23.30%		D(87,243,43)	[16]
Proximal DVT	65.10%		D(87,243,43)	[16]
VTE-related death	11.50%		D(87,243,43)	[16]
*Type of bleeding events*				
*Treatment phase*				
Dabigatran				
ICH	1.80%		D(2,22,85)	[8,9]
Other MB	20.20%		D(2,22,85)	[8,9]
Fatal MB (of other)	4.20%		Beta(α = 1,β = 23)	[8,9]
CRNMB	78.00%		D(2,22,85)	[8,9]
VKA				
ICH	2.10%		D(4,36,149)	[8,9]
Other MB	19.00%		D(4,36,149)	[8,9]
Fatal MB (of other)	5.00%		Beta(α = 2,β = 38)	[8,9]
CRNMB	78.80%		D(4,36,149)	[8,9]
*Secondary prevention*				
Dabigatran (RE-MEDY)				
ICH	2.50%		D(2,11,67)	[10]
Other MB	13.80%		D(2,11,67)	[10]
Fatal MB (of other)	0.00%		Fixed	[10]
CRNMB	83.80%		D(2,11,67)	[10]
Dabigatran (RE-SONATE)				
ICH	0.00%		Fixed	[10]
Other MB	5.60%		D(2,34)	[10]
Fatal MB (of other)	0.00%		Fixed	[10]
CRNMB	94.40%		D(2,34)	[10]
VKA				
ICH	2.80%		D(4,21,120)	[10]
Other MB	14.50%		D(4,21,120)	[10]
Fatal MB (of other)	4.00%		Beta(α = 1,β = 24)	[10]
CRNMB	82.80%		D(4,21,120)	[10]
*Other probabilities*				
Disabled from ICH	65.30%		Beta(α = 90.8,β = 48.2)	[15]
Probability of IHD after MI and UA	14%		Beta(α = 19,β = 116)	[17]
Cumulative incidence of CTEPH at 2 years in PE patients	3.80%		Beta(α = 7,β = 184)	[18]
Probability of CTEPH (per cycle)	0.16%			[18]
5 years cumulative incidence of severe PTS	8.10%		Beta(α = 43,β = 485)	[19]
Probability of severe PTS (per cycle)	0.14%			[19]
rVTE after therapy discontinuation	39.90%	35.40%–44.40%	Normal (SE = 0.02)	[16]
*Discontinuation probabilities (per cycle)*				
Treatment phase				
Dabigatran	2.09%		Fixed	[8,9]
VKA	1.91%		Fixed	[8,9]
Secondary Prevention				
Dabigatran	1.00%		Fixed	[10]
VKA	0.97%		Fixed	[10]

Furthermore, the probabilities of having a fatal VTE, non-fatal PE, proximal DVT, or distal DVT, were based on the incidences of these events in the aforementioned trials, and they were modelled to be conditional on having a VTE event. Similarly, the probabilities of having an ICH, other MB, fatal MB (including ICH) or CRNMB were conditional on having a MCRB. The proportion of ICH leading to permanent disability was assumed to be 65.3% [15].

Beyond the duration of the anticoagulant treatment, the lifetime probability of rVTE was calculated from the assumed 10-year cumulative incidence of 39.9% [16], assuming a constant hazard. The risk of bleeding after treatment discontinuation was assumed at zero for simplification, as the risk of bleeding will be equal in both arms and will not impact incremental QALYs. Probabilities of MI, UA and dyspepsia were estimated from the dabigatran trials [8–10]. For the treatment followed by secondary prevention, probabilities of MI, fatal MI and UA were calculated as the sum of probabilities in the treatment and secondary prevention trials. Events were assumed to occur at a constant rate during the trial follow-up. For simplicity, events were assigned to occur at the midpoint (i.e., three months). Additionally, we assumed 14% of MIs and UAs would lead to IHD [17].

The rate for CTEPH for index PE was estimated at 3.8% for two years [18]. For patients experiencing non-fatal rPE events, the risk of CTEPH was applied monthly up to 2 years [18].

Published evidence suggests that mild PTS has little detrimental effect on HRQoL [6], therefore, the model included only severe PTS. For all patients in index DVT, the 5-year rate of PTS was estimated to be 8.1% at model start [19]. A monthly probability of PTS subsequent to non-fatal rDVT events was applied up to 5 years [19]. Finally, the probability of death due to other causes was obtained from Statistics Netherlands.

### Utilities

Table 2 summarises the utilities used in the model. Patients were assigned baseline age- and gender-specific utilities derived from the general Dutch population [20]. These estimates formed the baseline from which the utility decrements associated with VTE, bleeding and other adverse events were subtracted.

**Table 2:** Utility parameters applied in the model. CRNMB = clinically relevant non-major bleed event; DVT = deep vein thrombosis; ICH = intracranial haemorrhage; LMWH = low molecular-weight heparin; MB = major bleed; MI = myocardial infarction; PE = pulmonary embolism; CTEPH = chronic thromboembolic pulmonary hypertension; PTS = post thrombotic syndrome.

Parameter	Value	Distribution	Reference
Baseline utilities			
Age 18–24 years (weight for males, females)	0.976, 0.925	Fixed	[20]
Age 25–34 years (weight for males, females)	0.945, 0.907	Fixed	[20]
Age 35–44 years (weight for males, females)	0.953, 0.917	Fixed	[20]
Age 45–54 years (weight for males, females)	0.902, 0.877	Fixed	[20]
Age 55–64 years (weight for males, females)	0.913, 0.866	Fixed	[20]
Age 65–74 years (weight for males, females)	0.878, 0.894	Fixed	[20]
Age ≥ 75 years (weight for males, females)	0.910, 0.787	Fixed	[20]
Disutility of index and recurrent DVT ^c^	0.250	Normal (SE = 0.0054)^a^	[11]
Disutility of index and recurrent PE ^c^	0.250	Normal (SE = 0.0152)^a^	[11]
Disutility of ICH or other MB ^d^	0.130	Gamma (α = 100, β = 0.001)	[11]
Disutility of disabled from ICH ^f^	0.380	Gamma (α = 16, β = 0.024)^b^	[21]
Disutility of CRNMB ^d^	0.040	Gamma (α = 100, β = 0.0004)	[11]
Disutility of MI ^d^	0.063	Gamma (α = 22.57, β = 0.003)	[22]
Disutility of Angina ^d^	0.085	Gamma (α = 40.40, β = 0.002)	[22]
Disutility of Dyspepsia ^e^	0.040	Gamma (α = 16, β = 0.003) ^b^	[23]
Disutility of CTEPH ^d^	0.440	Gamma (α = 16, β = 0.028) ^b^	[24]
Disutility of severe PTS ^f^	0.070	Gamma (α = 39.22, β = 0.002)	[6]

^a^ Change in mean from baseline to 3 months. In the probabilistic analysis, the mean baseline and 3-month value were individually sampled from normal distributions defined by the mean and standard error (standard error was calculated from the standard deviation and N) and the difference calculated for each simulation.

^b^ Variance was not reported; the standard error is assumed to be 25% of the mean.

^c^ The duration of disutility was assumed to be 6 weeks similarly to the previously published study.

^d^ A disutility is applied in the month of the event. Specifically, the duration of the impact of UA and MI on HRQoL was assumed to be 3 months.

^e^ The disutility applied is assumed to last for the duration of treatment.

^f^ A disutility is applied for the remaining lifetime.

Utility decrements associated with index and rVTE, MB and CRNMB were based on a meta-analysis of EQ-5D data collected in the RE-COVER and RE-COVER II trials and applied in the model similarly to the previously published study [11].

Utility decrements following the occurrence of other adverse events (i.e. MI, UA, dyspepsia, disabled from ICH, CTEPH, and severe PTS) were derived from the published studies and applied additively for a specific time interval in the model [6,21–25].

### Costs

In the base-case analysis, all costs were collected from a societal perspective, therefore, both direct (inside and outside healthcare) and indirect costs were included (Table 3). Direct costs inside healthcare included the costs related to: drugs, visits to general practitioner (GP), administration, INR-monitoring and event-related resource use. Costs of dabigatran (price per defined daily dose at 2x 150mg), VKAs and LMWHs were taken from the official Dutch price list (Z-index) [26]. Importantly, the price of dabigatran extracted from Z-index is established for other registered indications of dabigatran in the Netherlands (i.e. prevention of VTE in patients who have undergone elective total hip replacement surgery or total knee replacement surgery, and prevention of stroke and systemic embolism in non-valvular atrial fibrillation). Acenocoumarol and phenprocoumon are the only VKAs registered in the Netherlands, therefore, the cost of VKAs was estimated as a weighted average of the costs of those drugs based on their usage in the Netherlands (80%:20%, respectively) [27]. The cost of LMWHs was assumed as a weighted average cost of enoxaparin, dalteparin, tinzaparin and nadroparin [26]. All treatment alternatives were assumed to have a cost of one initial GP visit in the first month and one follow up visit in the 4^th^ month of a treatment.

**Table 3:** Cost parameters applied in the model. VKA, vitamin K antagonists; LMWH, low molecular weight heparins; INR, international normalised ratio; MCRB, major or clinically relevant bleeding; DVT, deep vein thrombosis; PE, pulmonary embolism; CRNMB = clinically relevant non-major bleed event; ICH = intracranial haemorrhage; MB = major bleed; MI = myocardial infarction; UA, unstable angina; CTEPH = chronic thromboembolic pulmonary hypertension; PTS = post thrombotic syndrome GP, general practitioner.

Cost parameters	Average cost (2013, €)	Range^a^	Reference
Medication, administration and monitoring costs			
VKA (daily)	0.04	0.03–0.05	[26]
Dabigatran (daily)	2.30	Fixed	[26]
LMWH (daily)	10.65	7.99–13.31	[26]
LMWH at home, self-injection (one-off training)	16.77	9.59–25.93	[29]
LMWH at home, nurse injection (per day after discharge)	17.50	10.00–27.05	[29]
LMWH, administration in clinic (per day after discharge) incl. travel costs	16.54	9.45–25.57	[29]
LMWH at home, self-injection (domiciliary care)	6.74	3.85–10.43	[29]
GP visit	30.54	17.46–47.22	[14]
INR-control self-management initial monthly cost	90.46	51.71–139.88	[14,33]
INR-control cost incl. travel costs (per visit) ^b^	12.54	7.17–19.38	[14,33]
INR-control self-management (monthly)	12.29	7.03–19.01	[14,33]
Events costs			
DVT	1,187.23	679–1,836	[32]
PE	4,221.01	2,413–6,527	[32]
ER visit	167.28	96–259	[32]
Chest x-ray	156.15	89–241	[32]
Electrocardiogram	30	17–46	[32]
Acute ICH	32,754	18,722–50,646	[21]
ICH direct mild (annually)	2,367.97	1,354–3,662	[21]
ICH direct moderate (annually)	18,268	10,442–28,247	[21]
ICH direct severe (annually)	23,353	13,348–36,110	[21]
MB	4,969	2,840–7,683	[32]
CRNMB ^c^	31	17–47	[14]
PTS (year 1)	25,073	14,331–38,769	[32]
PTS (year 2) ^d^	61	35–94	[14]
MI acute	5,021	4,936–5,106	[34]
MI follow up (monthly)	97	55–150	[35]
UA	5,351	5,236–5,467	[36]
Dyspepsia ^e^	0.69	0.39–1.07	[26]
CTEPH acute ^f^	7,121	4,070–11,011	[37]
CTEPH follow up (monthly)	84	48–130	[37]
Indirect costs			
Productivity loss age group 55–60 (per hour) ^g^	31	17–47	[14]
Productivity loss age group 60–65 (per hour) ^g^	23	13–36	[14]
ICH informal care mild (annually)	12,369	7,070–15,462	[38]
ICH informal care moderate (annually)	16,345	9,343–25,274	[38]
ICH informal care severe (annually)	20,322	11,616–31,422	[38]

^a^ Cost estimates that were available only as single point estimates, were assumed to follow a log-normal distribution with a coefficient of variation equal to 0.25.

^b^ Travel costs of patients included only in the base-case.

^c^ Assumed to be equal to the cost of a GP visit.

^d^ Assumed to be equal to the cost of two GP visit.

^e^ Assumed the cost of Omeprazol 20mg.

^f^ Based on the study by Mayer et al, pulmonary endarterectomy is applied to 56.8% of cases.

^g^ One hour of productivity loss costs was estimated as a weighted average cost for employed and non-employed population in the Netherlands in the specific age group.

The cost of administration of LMWHs was estimated to reflect the costs of administration in hospital and at home. For patients receiving LMWHs in hospital, the costs of administration were adjusted for the percentage of patients and time they spent being hospitalized for DVT and for PE [28]. The costs of administration at home accounted for the costs of self-injection and costs for patients requiring a nurse visit for injection (Table 3)[28,29].

The costs of INR-monitoring reflected the costs of monitoring handled by thrombotic services and costs for patient self-management (Table 3). In the Netherlands, self-management is applied by 14.9% of patients on treatment with VKAs. Therefore, the costs of initial training for self-management and monthly follow up costs associated with the rental of equipment were applied for this patient population (Table 3). Resource use associated with INR-monitoring at thrombotic services (i.e. number of visits) was based on the annual medical reports from the Dutch thrombotic services [30][31]. In particular, in the first month of a treatment, the cost of INR-monitoring by thrombotic services reflected an average of 5.5 visits to thrombotic services. In follow up months (2^nd^ until 6^th^ month), the cost of 1.4 visits per month was assumed [30][31]. For the application of VKAs longer than 6 months (i.e. secondary prevention phase), the costs of INR-monitoring by thrombotic services reflected an average of 1.4 visits per month beyond the initial 6-month period [30,31]. Moreover, direct costs outside healthcare (i.e. travel costs) were attributed to the nurse visits for injection of LMWHs. Acute care costs associated with clinical events (e.g. DVT, PE, ICH, other MB, CRNMB, PTS, CTEPH, MI, UA, and dyspepsia) were adopted from previous costing studies conducted in the Netherlands. Patients surviving acute ICH, MI, PTS and CTEPH were assigned with long-term maintenance costs.

Indirect costs outside healthcare included: productivity loss costs, caregiver time costs for patients experiencing ICH and travel costs for the visits of patients to thrombotic services. A 2-hour productivity loss costs were assumed for all INR-monitoring visits to thrombotic services and all GP-related visits. Additionally, productivity loss costs associated with hospitalizations due to DVT (0.63 days), PE (7 days) and MI (5.6 days) were included. The number of productivity loss hours for each of the aforementioned hospitalizations was estimated in order to account for regular working hours (8 hours per day) was corrected for the weekends and the labour-time elasticity of production according to the friction costing method [14,32].

### Sensitivity analyses

Univariate sensitivity analyses were performed to identify the key determinants of CE by varying parameters individually over the ranges derived from their 95% confidence intervals. Where confidence intervals and standard deviations of parameters were unavailable, the standard error was assumed to be 25% of the mean. The exceptions were made when varying discount rates which were varied between 0 and 5%, and the number of days on treatment with LMWHs which were varied between 5 and 9 days. The results were defined in terms of incremental cost per QALY and are presented diagrammatically in the form of a tornado diagram.

Additionally, a probabilistic sensitivity analysis (PSA) was performed to assess the robustness of the findings by performing 5000 simulations to generate ICERs in which event risks and HRs, costs and utilities were simultaneously varied randomly within their ranges. HRs of the even rates were sampled from a normal distribution on the log scale and other probabilities were sampled from a beta distribution. The related distribution of the type of recurrent event were sampled from a Dirichlet distribution. Costs were sampled from a gamma distribution. For utilities, a gamma distribution was used, except for utilities assigned to DVT and PE, for which a normal distribution was used. Results from the PSA were plotted on a CE plane.

### Scenario analyses

To investigate the impact of applying dabigatran under different decision making settings seven scenario analyses were conducted. First scenario compared the use of dabigatran and VKAs for treatment and secondary prevention in high risk patients from the healthcare provider perspective. Second scenario compared the use of dabigatran to VKAs for up to 6 months of treatment only. Third scenario assessed the use of anticoagulants for up to 18 months of secondary prevention only (not considering the preceding treatment duration). In the fourth scenario, the use of dabigatran for up to 6 months of secondary prevention (not considering the preceding treatment duration) was compared to placebo. Here, study population simulated the profile of the patients in the RE-SONATE trial, i.e. low risk patients (i.e. patients for whom the need for secondary prevention is at equipoise [10]). Data from the RE-SONATE trial were the main sources used to estimate the transition probabilities between the health states in the model in this scenario. In the fifth scenario disutilities associated with VKA use were applied to the model. Furthermore, as an alternative source the costs of ICH as applied by Ten Cate-Hoek et al. was applied to the model (scenario 6), lacking however the detailing of separate cost figures for mild, moderate and severe ICH [32]. In the last scenario the transition probabilities of ICH were reduced to half of its base-case values.

All assumptions used in the model are summarized in Table 4.

**Table 4:** Overview of assumptions used in the model.

• The duration of LMWH use in the dabigatran and VKA arm was assumed to be 5 and 9 days respectively, based on the RE-COVER trials • Only patients who remained in the index state after 6 months initial treatment were signed up for 18 months extended treatment, reflecting patient profiles from the RE-MEDY trial • The model was restricted to a maximum of two rVTEs per patient • After a first rVTE, patients from both treatment arms were assumed to stop the initial treatment and initiate or reinitiate a 6 months standard treatment course of LMWHs, followed by VKAs • No differentiation between high and low risk MCRB • The model was restricted to a maximum of two MBs per patient during the time on anticoagulation: one event could be experienced during treatment phase with study medication and one event during LMWHs/VKAs re-treatment • CRNMB could occur at every model cycle while on anticoagulation • After a MB all patients discontinued anticoagulation treatment, having no further risk of bleeding • Mortality after chronic ischemic heart disease (IHD) is part of the population mortality in the model • Patients who experience PE are at risk of CTEPH • Patients who experience DVT are at risk of PTS • After treatment discontinuation due to any cause patients are still at risk of rVTE, but have no bleeding risk • Patients in any of the health states are at risk to die from other causes • Only the risk of severe PTS is included in the model • The cost of LMWHs was assumed as a weighted average cost of enoxaparin, dalteparin, tinzaparin and nadroparin • All treatment alternatives were assumed to have a cost of one initial GP visit in the first month and one follow up visit in the 4^th^ month of a treatment • 2-hour productivity loss costs were assumed for all INR-monitoring visits to thrombotic services and all GP-related visits

## Results

In the *base-case*, in a hypothetical cohort of 10,000 patients with a VTE event followed over their lifetime starting at age 54.7 years, dabigatran averted 720 MCRBs compared with VKAs but resulted in an additional 86 rVTEs, and 65 MIs (Table 5). A comparable number of PTS, CTEPH and UA was observed in both dabigatran and VKAs treatment arms. Dabigatran was associated with a projected discounted quality-adjusted life expectancy of 19.187 QALYs compared with 19.154 QALYs for patients receiving VKAs.

**Table 5:** Recurrent VTE, bleeding complications and other adverse events and related costs within a hypothetical patient population of 10,000 subjects receiving dabigatran and VKA over a lifetime horizon. VKA, vitamin K antagonists; VTE, venous thromboembolism; DVT, deep vein thrombosis; PE, pulmonary embolism; r, recurrent; LMWH, low molecular weight heparin; CRNMB = clinically relevant non-major bleed event; ICH = intracranial haemorrhage; MB = major bleed; MI = myocardial infarction; CTEPH = chronic thromboembolic pulmonary hypertension; PTS = post thrombotic syndrome; UA, unstable angina; INR, international normalised ratio.

	Dabigatran		VKA	
	Number of events	Costs p.p. (undiscounted)	Number of events	Costs p.p. (undiscounted)
Index VTE	10,000	€2,142	10,000	€2,142
All recurrent VTE	13,471		13,384	
Recurrent non-fatal VTE	11,959		11,871	
Non-fatal DVT	8,761	€1,071	8,713	€1,065
Non-fatal PE	3,198	€1,592	3,158	€1,570
VTE-related death	1,512	€0	1,513	€0
All MCRBs	1,351		2,071	
Non-fatal MCRBs	1,342		2,052	
ICH	28	€1,876	47	€3,262
Other MBs	230	€118	339	€177
CRNMBs	1,084	€9	1,665	€14
Deaths from bleeding	9	€0	19	€0
MI	86	€93	21	€23
UA	23	€23	23	€23
Dyspepsia	682	€0.05	112	€0.01
PTS	1,294	€3,482	1,290	€3,471
CTEPH	243	€667	242	€662
Medication				
Investigational treatment		€1,315		€24
LMWHs, index event		€71		€110
Re-treatment recurrent event, VKA		€12		€12
Re-treatment recurrent event, LMWHs		€152		€152
Monitoring and administration				
INR-monitoring, GP visits, administration and productivity loss		€167		€2,507
Administration of LMWHs		€43		€81
Re-treatment with VKA for recurrent event:INR-monitoring, GP visits, administration and productivity loss		€799		€794
Administration of LMWHs		€131		€130

Costs allocation across different categories indicated that costs associated with handling rVTE, bleeding and other adverse events were the major contributors to the total expenditures. In VKAs treatment arm, these costs were higher compared to dabigatran arm (€12,409 vs €11,074). Expenditures for event-related costs were followed by monitoring, GP visit, productivity losses and administration costs which were higher with VKAs compared to dabigatran (€3,512 vs. €1,140), and the total drug costs that were higher with dabigatran than VKAs (€1,550 vs. €298). Finally, accounting for all the aforementioned cost categories resulted in the total lifetime costs varied from €12,133 per person for VKAs to €10,209 per person for dabigatran at a discount rate of 4%. In total, savings of €1,924 and an additional 0.0339 discounted QALYs per patient where observed when applying dabigatran compared to VKAs (Table 6).

**Table 6:** Results of the base-case and scenario analyses. VKA, vitamin K antagonists; ICER, incremental cost-effectiveness ratio; QALY, quality adjusted life year; LY, life year.

Base-case: 6 months treatment + 18 months secondary prevention (societal perspective)			
	Dabigatran	VKA	Difference
Discounted LYs	22.053	22.025	0.0282
Discounted QALYs	19.187	19.154	0.0336
Costs (€) undiscounted	13,637	15,805	-2,168
Costs (€) discounted	10,071	11,668	-1,598
ICER (€/ LYs)	Cost-saving		
ICER (€/ QALYs)	Cost-saving		
Scenario 1: 6 months treatment + 18 months secondary prevention (healthcare provider perspective)			
Discounted LYs	22.053	22.025	0.0282
Discounted QALYs	19.187	19.154	0.0336
Costs (€) undiscounted	12,115	12,307	192
Costs (€) discounted	9,051	8,978	73
ICER (€/ LYs)	2,575		
ICER (€/ QALYs)	2,158		
Scenario 2: 6-months treatment (societal perspective)			
Discounted LYs	21.924	21.907	0.0170
Discounted QALYs	19.083	19.063	0.0197
Costs (€) undiscounted	11,974	13,024	-1,050
Costs (€) discounted	8,795	9,615	-819
ICER (€/ LYs)	Cost-saving		
ICER (€/ QALYs)	Cost-saving		
Scenario 3: 18-months secondary prevention in high-risk patients (societal perspective)			
Discounted LYs	22.044	22.030	0.0144
Discounted QALYs	19.248	19.230	0.0180
Costs (€) undiscounted	10,101	11,324	-1,224
Costs (€) discounted	6,535	7,370	-835
ICER (€/ LYs)	Cost-saving		
ICER (€/ QALYs)	Cost-saving		
Scenario 4: 6-months secondary prevention vs no treatment* in patients for whom the need for secondary prevention is at equipoise (societal perspective)			
Discounted LYs	21.950	21.950	0.0003
Discounted QALYs	19.169	19.165	0.0035
Costs (€) undiscounted	7,945	7,847	98
Costs (€) discounted	4,923	4,807	117
ICER (€/ LYs)	429,111		
ICER (€/ QALYs)	33,379		
Scenario 5: treatment disutility VKA included (societal perspective)			
Discounted Lys	22.053	22.025	0.0282
Discounted QALYs	19.187	19.129	0.0583
Costs (€) undiscounted	13,637	15,805	-2,168
Costs (€) discounted	10,071	11,668	-1,598
ICER (€/ LYs)	Cost-saving		
ICER (€/ QALYs)	Cost-saving		
Scenario 6: costs ICH based on Ten Cate-Hoek et al.			
Discounted Lys	22.053	22.025	0.0282
Discounted QALYs	19.187	19.154	0.0336
Costs (€) undiscounted	13,166	14,998	-1,821
Costs (€) discounted	9,800	11,187	-1,386
ICER (€/ LYs)	Cost-saving		
ICER (€/ QALYs)	Cost-saving		
Scenario 7: Incidence ICH events reduced to half of its base-case values			
Discounted Lys	22.053	22.027	0.0266
Discounted QALYs	19.193	19.166	0.0269
Costs (€) undiscounted	12,951	14,426	-1,475
Costs (€) discounted	9,672	10,867	-1,195
ICER (€/ LYs)	Cost-saving		
ICER (€/ QALYs)	Cost-saving		

*placebo risks from the clinical trial being used

### Sensitivity analyses

**Fig 2:**
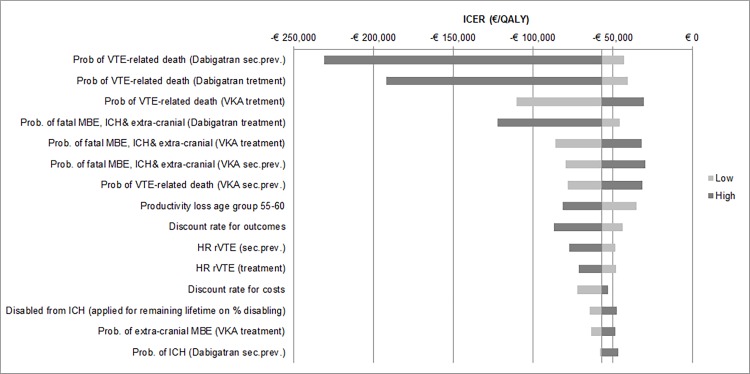
**Tornado diagram of ICERs from sensitivity analyses for dabigatran vs. vitamin-K antagonists,** illustrating the impact of varying each of input parameters on the ICER while holding all the other model parameters fixed. Light grey bars show the influence of using the upper limit and dark grey bars that of the lower limit of the input parameters investigated. The solid vertical line represents the base case incremental costs per QALY for dabigatran compared to VKA. Horizontal bars indicate the range of incremental costs per QALY obtained by setting each variable to the values shown while holding all other values constant. ICER, incremental cost-effectiveness ratio; QALY, quality adjusted life year; VKA, vitamin K antagonists; VTE, venous thromboembolism; r, recurrent; ICH = intracranial haemorrhage; MBE = major bleeding event; HR, hazard ratio.

**Fig 3:**
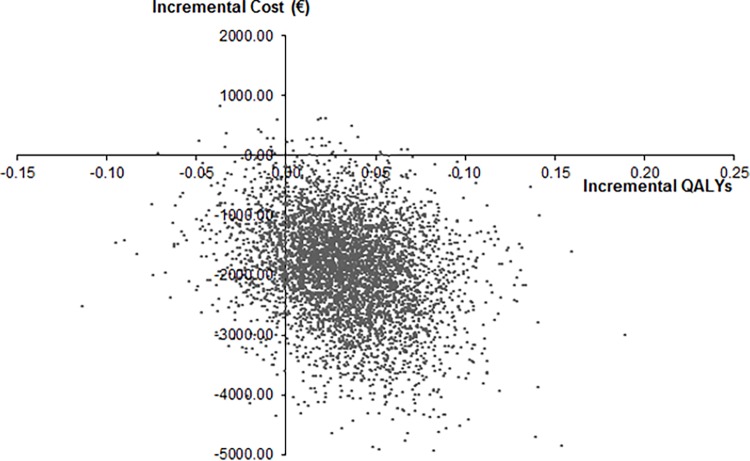
Incremental cost-effectiveness plane.

The results of univariate sensitivity analyses for the top 15 parameters by the order of influence they have to the ICERs are presented in the form of a tornado diagram (Fig 2). Specifically, the ICER was mostly influenced by variations in the probability of VTE-related death, probability of MCRBs-related and probability of ICH. The results of 5,000 iterations in PSA are presented through an incremental CE plane in Fig 3. The probability that dabigatran is cost-effective at a willingness-to-pay (WTP) threshold of €20,000/QALY was 98.1%, and 87.2% when ICERs with negative quality of life were counted as not cost-effective. 87.2% of the simulations are located in the second quadrant of the CE plane, in which costs are negative and QALYs are positive. At a WTP threshold of €50,000/QALY dabigatran is cost-effective in 87.4% of the simulations (ICERs with negative QALYs included as not cost-effective).

### Scenario analyses

The results of the scenario analyses are presented in Table 6. In the scenario comparing dabigatran to VKAs for the treatment, and in the one comparing them for the secondary prevention in high-risk patients, dabigatran remained cost-saving. The scenario including the disutility associated with VKA treatment, as well as the last two scenarios concerning the costs and transition probabilities of ICH, also remained cost-saving. Comparing dabigatran to VKAs for treatment and secondary prevention from the healthcare provider perspective dabigatran was cost-effective with an ICER of €2,158 per QALY gained. Finally, in the scenario examining the prevention of recurrent VTE in patients with equipoise risk of recurrent VTE after completion of acute treatment, anticoagulation treatment with dabigatran compared to no treatment (placebo risks from the clinical trial being used), yielded an ICER of €33,379 per QALY gained.

## Discussion

Our base-case result from the decision analysis demonstrated dabigatran may be a cost–saving alternative to VKAs for the treatment and secondary prevention of VTE from the societal perspective. Patients on dabigatran gained an additional 0.0336 discounted QALYs over lifetime and savings of €1,598. The key drivers of the CE of dabigatran relative to VKA are based on its ability to reduce MCRBs as found in the RECOVER trials. Particularly, the use of dabigatran resulted in 720 less all MCRB events (i.e. 19 ICHs, 109 other MBs, 582 CRNMBs and 10 deaths from bleeding) in a cohort of 10,000 patients.

Results were sensitive to the probability of VTE-related death, MCRBs-related death and ICH, yet, they all indicated dabigatran to be cost-saving compared to VKAs. Moreover, the PSA showed that the likelihood of dabigatran being cost-effective at WTP threshold of €20,000 per QALY was 98.1%. This also included the cost-effective ICERs in the south eastern part of the CE plane, in which QALYs and costs are negative. Since a reduction of quality of life is not desirable, these ICERs might not be considered cost-effective. Therefore, the probability of being cost-effective at a WTP thresholds of €20,000/QALY and €50,000/QALY while not lowering the quality of life was 87.2% and 87.4%, respectively. For economic analyses determining the cost-effectiveness of preventive drugs, for example for vaccines, a WTP threshold of 50,000/QALY can be used [39]. In this economic evaluation dabigatran showed to be already highly cost-effective at a WTP threshold of €20,000/QALY, leading to only a marginal increase of being cost-effective with a higher threshold.

The results of the scenario analyses comparing dabigatran to VKAs for the treatment and secondary prevention in high-risk patients of VTE, were quite robust, all indicating dabigatran may be cost-saving alternative to VKAs. Changing the duration of anticoagulation to life long has only a marginal impact on the ICER (results not explicitly shown). Although ICH risks showed to be sensitive in the sensitivity analysis, the two scenarios concerning the costs and the transition probabilities of ICH showed to be still cost-saving, However, in the scenario examining the CE of anticoagulants for the treatment and secondary prevention from the healthcare provider perspective, dabigatran was shown to be a cost-effective alternative to VKAs with an ICER of €2,158 per QALY gained. Interestingly, although the variability in productivity loss costs showed an impact on the estimated ICER in the univariate sensitivity analyses, excluding these costs together with other indirect costs still led to highly cost-effective findings in the aforementioned scenario. Finally, in the scenario examining the prevention of recurrent VTE in patients who are at equipoise for anticoagulation treatment, treatment with dabigatran compared to placebo was estimated with an ICER of €33,379 per QALY and may be considered cost-effective at the proposed cost-effectiveness threshold of €50,000 per QALY gained in the Netherlands [39]. This finding reflects the higher total costs associated with greater number of MCRBs and drug costs in dabigatran treatment arm compared to placebo arm. We do note that anticoagulation treatment in this population is not established, current treatment guidelines leave the decision about treatment to the choice of physicians [2], despite projected potential lowering of rVTEs. Further confirmations of results might definitely be needed for this patient population before widespread use in clinical practice can be expected.

To our knowledge, this is the first study that examined the use of dabigatran compared to VKAs for the treatment and prevention of VTE in the Dutch setting. In terms of the economic consequences of using dabigatran compared to VKAs, our findings are similar to the ones by Braidy et al [40]. In this study a cost-minimisation analysis investigated the use of NOACs and VKAs for the prevention of VTE and stroke in patients with atrial fibrillation from the third-party payer perspective in an Australian setting [40]. Dabigatran was found to be dominant over VKA (cost savings at approximately $AUS40 per patient) in terms of cost of drug administration and therapeutic monitoring. Notably, a direct comparability between the two studies is hampered due to differences in the underlying patients’ characteristics, safety and effectiveness data used, country-specific cost estimates and study perspective.

Our study is confronted with several potential limitations. One limitation might be that the duration of initial treatment with LMWHs was assumed to be different in dabigatran and VKAs treatment arms in the base-case analysis (i.e. 5 and 9 days respectively). Notably, the duration of treatment with LMWHs was assumed to have no impact on the effectiveness of the follow up use of dabigatran and VKAs. We varied the duration of LMWHs treatment between 5 and 9 days for both treatment alternatives in univariate sensitivity analyses. The results remained robust to variability in the duration of LMWHs use. Furthermore, this study simulated the occurrences of all MCRBs further subdivided into ICHs, other MBs, CRNMBs and deaths from bleeding, however, the meta-analysis of the RE-COVER trials indicated that there was only a marginally significant reduction of MBs observed in the double-dummy period in dabigatran arm compared to VKA. Therefore, simulating the occurrences of MBs might overestimate the benefits in the dabigatran arm compared to VKA. Yet, acute coronary syndromes (i.e. MIs and UAs) were also modelled in this study although their incidence was only numerically higher with dabigatran compared to VKAs. A further potential limitation in our study concerns the assumption that patients in both treatment arms who experience a first recurrent VTE event would switch to a 6-months standard treatment course of LMWH followed by VKAs. This may not always be the case and patients might alternatively be switched to other NOACs. However, there are currently no available efficacy and safety data that could characterize such a switch.

A maximum of two rVTEs over the lifetime of the patients and two MBs during the anticoagulation treatment were modelled. This assumption may be considered conservative given that a better safety profile of dabigatran treatment would be associated with a lower number of MBs and consequent lower costs compared to treatment with VKAs. Another limitation concerns treatment discontinuation that was assumed for patients who experience a MB or ICH. In a real-life setting such a decision would likely be based on individual patient characteristics. Finally, given the lack of specific treatment recommendations for patients experiencing CRNMBs, the discontinuation of treatment due to CRNMB was not modelled. Notably, in daily practice, patients may discontinue with the treatment after a certain number of consequent CRNMBs. However, as dabigatran can now be reversed by idarucizumab (Praxbind), we postulate that the manageability is not the major contributor of severity and outcome of the bleed, but rather the underlying disease and other disorders [41]. Notably, given all limitations listed, further investigations could be guided by a formal value-of-information (VOI) analysis. The current version of the model does not yet foresee in this option and we felt that the current analysis would hardly benefit from incorporating it. Firstly and most notably, strongly differing from UK’s NICE, the Netherlands lack a formal willingness-to-pay threshold for a QALY [42], crucially hampering any straightforward interpretation of and inferencing from the VOI. Secondly, part of the need for further data generation is already addressed via initiation of the RECOVER registry (phase IV clinical trial), designed to evaluate dabigatran for DVT and PE in real life [8,9]. Finally, regarding uncertainty and within the framework of a general model validation for NICE, we previously already analyzed inserting alternative distributions in the PSA, showing that this had only limited impact on the results of the model [43]. In conclusion, from a societal perspective, this modelling study suggests that the use of dabigatran for treatment and secondary prevention of VTE is likely to be a cost-saving alternative to VKAs in the Netherlands. Importantly, even when the comparison between dabigatran and VKAs was assessed from the healthcare provider perspective, dabigatran remained highly cost-effective with an ICER of €2,158 per QALY gained as compared to a minimal WTP threshold €20,000 per QALY. Ergo, in addition to some established advantages of dabigatran (e.g. better safety profile than VKAs; excludes the need for INR-monitoring), our study estimated the long-term economic benefits associated with its use. Yet, it must be acknowledged that such benefits in a “real life” setting are still to be proven. Finally, given that dabigatran is the second NOAC registered in Europe for the treatment and secondary prevention of VTE and further are to be expected, further investigations are needed to estimate comparative effectiveness and CE among the individual NOACs and as a class effect.

## Supporting Information

S1 Fig VTE Tx Model v2.0_update v13_js_121014_EDLJ.xlsm. The Microsoft Excel model of Dabigatran for the treatment and secondary prevention of venous thromboembolism; a cost-effectiveness analysis for the Netherlands.
(XLSM)
